# Depth-Variant Deconvolution Applied to Widefield Microscopy for Rapid Large-Volume Tissue Imaging

**DOI:** 10.21203/rs.3.rs-6710731/v1

**Published:** 2025-06-06

**Authors:** Daniel D. Lee, Kevin A. Telfer, Mark. A. J. Koenis, Yim K. Lee, Kevin W. Namink, Brian T. Saunders, Heyun Lee, Hailey K. Kelley, Heather S. Ruiz, Joseph P. Gaut, Gwendalyn J. Randolph, Bernd H. Zinselmeyer

**Affiliations:** 1 Department of Pathology & Immunology, Washington University School of Medicine, Saint Louis, MO, USA; 2 Scientific Volume Imaging B.V., Hilversum, Netherlands; 3 Independent Scholar, Saint Louis, Missouri, USA; 4 Zeiss Microscopy, Saint Louis, Missouri, USA

## Abstract

Innovations in 3D tissue imaging have revolutionized research, but limitations stemming from lengthy protocols and equipment accessibility persist. Classical widefield microscopy is fast and accessible but often excluded from 3D imaging workflows due to its lack of optical sectioning. Here we combine tissue clearing with a depth-variant deconvolution approach customized for large-volume widefield imaging to achieve subnuclear axial resolution in tissues to a depth of 500 μm. We illustrate the utility of this method in a mouse model of ileitis and to gain a 3D perspective in thick brain slices from a mouse model of cerebral amyloid angiopathy, where we resolved large and small blood vessels, including those with amyloid deposits, attaining resolution that compared favorably to tile-scanning confocal microscopy. Finally, we sought to leverage our approach to allow for richer pathological evaluation of human kidney biopsies. Our approach produced hundreds of consecutive z-planes in five minutes of imaging for 3D visualization of winding arterioles feeding into glomeruli. This 3D perspective afforded straightforward identification of atrophic tubes in fresh kidney biopsies prepared in 2 hours to simulate the time-constrained evaluation of donor kidneys for transplant suitability. Having achieved subnuclear z-resolution in sections hundreds of microns thick, widefield microscopy coupled to robust deconvolution now emerges as an accessible and viable method to gain 3D insight in research or clinical pathological evaluations.

## Introduction

Several methods for tissue preparation and refractive index matching to achieve rapid, even, and thorough tissue transparency have been developed. These include CUBIC^1^, DISCO^2^, EZClear^3^, and ADAPT-3D^4^. We wondered if these improvements to image processing could be combined with cost-effective, fast, and accessible 3D imaging carried out using widefield epifluorescent microscopy. At first glance, widefield epifluorescent microscopy might seem outdated and the least likely to find utility at the forefront of 3D imaging due to diffuse noise inherent to widefield microscopy that obscures and blurs signal. Widefield microscopy does not have the advantage of confocal, two-photon, or light-sheet microscopy, where collection of light in a tissue sample is limited to a defined layer of tissue by a pinhole, the temporal compression of photons, or an arrangement of lenses that creates spatial illumination, respectively. However, given that widefield epifluorescent microscopes are often more accessible and user friendly than other sophisticated systems, we reasoned that improving their utility in deep 3D imaging could have advantages. Moreover, a widefield system with precision z-axis control creates an opportunity to collect x-, y-, and z-plane data with widefield systems. However, much of the emitted light collected will be out of focus as it spreads through the many z-layers of tissue, to the point where widefield microscopy has largely been disregarded for providing meaningful axial information. Correction of this blurring to reconstruct a sharp image that places detected light at its point of origin can be estimated, or deconvolved, with a point spread function. A point spread function (PSF) is a functional shape that describes the diffraction of light through an optical system^5^. It thus considers properties of the system. Both open source (e.g., DeconvolutionLab2 or DeconWolf) and commercial systems (e.g., AutoQuant, Scientific Volume Imaging Huygens) ably estimate the diffraction of light in deconvolution modalities ^6–8^. However, advances in 3D deconvolution using widefield microscopy have mainly been demonstrated on samples that are little more than 1-2 cell layers in thickness or are mostly applied to other modalities like light-sheet imaging^9^.

Application of deconvolution to thicker tissues or cell layers requires PSFs that account for and vary with depth of the sample. With recent advances in machine learning algorithms and improvements to computer hardware (e.g., graphics processing unit), there have been efforts to scale deconvolution to improve depth-variant analysis. However, depth-variant deconvolution remains a challenge, and it has not yet been demonstrated successfully in large-volume 3D widefield imaging. While tissue thickness is one challenge, widefield epifluorescent microscopy adds the additional challenge of a large diffraction limit that arises from low numerical apertures on objective lenses in combination with large spherical aberration. Spherical aberration is a phenomenon where rays of light do not converge toward a focused point due to a mismatch in refractive indices as light passes through multiple imaging depths^10^.

To overcome the challenges of unrestrained light collection in widefield microscopy that are accentuated in deep tissue imaging and achieve rapid start-to-finish large-volume 3D tissue imaging with an accessible widefield system, we herein describe how we adapted and combined the commercially available software product Scientific Volume Imaging Huygens, which starts with depth-variant PSFs, with optimized image acquisition using a turn-key commercial microscope. We demonstrate successful depth-variant deconvolution of images acquired during widefield microscopy through a 0.5-mm tissue depth, yielding results comparable to the quality of confocal images in a fraction of the time. We go on to further expand the tissue volume that could be analyzed by developing a protocol for x- and y- tiling of thick stacks of depth-variant deconvoluted images to reconstruct significantly larger tissue areas in a volume of approximately 1 cm^3^. These technical advances, allowed us to visualize cerebral amyloid angiopathy (CAA) in 3D, a disease characterized by Aβ plaque deposition along brain vasculature. Finally, we demonstrate the potential of this approach for application in a mock clinical scenario wherein biopsies of human donor kidneys are evaluated for transplant suitability, typically within a constrained time frame^11^. The speed and quality of our approach allowed for robust 3D information to be collected in a rapid time frame to assess the patency and characteristics of kidney glomeruli.

## RESULTS AND DISCUSSION

### Key elements of a turn-key epifluorescent widefield microscope for large-area 3D imaging

To achieve high axial resolution in thick tissue, we configured a system optimized for rapid acquisition of in-focus signal along the axial z-axis. We used a standard base for the microscope with an electronically controlled z-drive fitted with a 20× immersion objective (NA 1.0, the highest resolution on market) that has a spherical aberration collar, enabling lossless sampling of focal planes at a Nyquist rate of less than 1 μm apart in axial z. While high-NA lenses typically have limited working distances, our selected objective featured a 6.4-mm working distance, optimal for deep tissue imaging. This configuration permitted collection of extensive in-focus axial information at greater depths than would be possible in less ideal configurations.

**Figure 1.**
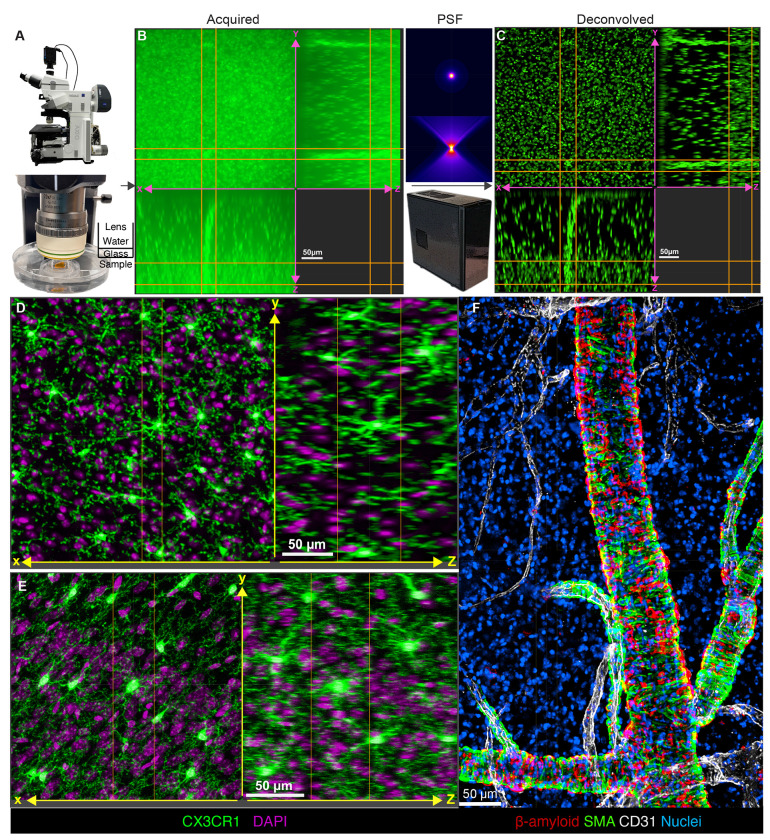
Depth variant deconvolution of thick image volumes acquired with a widefield microscope achieves sub-nuclear axial resolution for 3D visualization. (A) Standard epifluorescent microscopy build with 20X immersion lens (Carl Zeiss AG, NA 1.0, working distance of 6.4 mm) (top). Optical path for a refractive index matched tissue mounted under glass coverslip with 20X lens that is immersed in water (bottom). (B) Extended display showing raw image of nuclei in a cleared mouse brain labeled with anti-Histone-H3-ATTO488 (green) acquired on widefield microscope before computational deconvolution. (C) Nuclei in cleared brain after deconvolution with depth-variant point spread functions calculated using Huygens Software (Scientific Volume Imaging) and visualized in the axial dimension with Imaris software. (D) Extended display showing resolution of fine microglial pseudopods labeled by Cx3cr1-tdTomato (green) and nuclear staining with anti-Histone-H3-ATTO488 (magenta) captured using epifluorescent widefield microscopy with 20X (NA 1.0) lens; (E) Extended display of microglia as in (D) but captured using line-scanning confocal microscopy at a similar pixel resolution. (F) Maximum intensity projection of ~200 microns of 5XE4 leptomeninges attached to the brain surface that were stained for Aβ (red) to label CAA plaques along arterioles (smooth-muscle actin: green, CD31-positive vessels: gray), imaged using epifluorescent widefield microscopy with 5X objective lens (NA 0.16, air), and deconvolved.

To image cleared samples, we engineered a sample chamber with the objective lens immersed in water and separated from the refractive index matched sample by a glass coverslip (Fig. 1A). Spherical aberrations from refractive index mismatches in the optical path were mitigated using the lens’ correction collar. A 5-bandpass filter combined with a 7-LED light source enabled rapid, switch-free excitation across channels, a feature that improved upon the inherent speed benefits of camera-based imaging. At the time of this manuscript, the total cost of our widefield system was under $125,000 and required no service contracts.

### Axial nuclear resolution by deconvolution with depth-variant point spread functions

Using the widefield microscope build described above, over a 5-minute period, we collected hundreds of z-slices at 0.8-μm step intervals from mouse brain tissue that had been processed using the ADAPT-3D methodology^4^ and stained with an ATTO 488-conjugated anti-histone H2A-H2B heterodimer nanobody. Whereas tissue processing enabled collection of emitted light to a depth of 500 μm, the acquired z-slices were observed as a mixture of blurred emitted light from nuclei and out-of-focus light derived from nuclei in other neighboring z planes (Fig. 1B). To remove out-of-focus light from the image, we used the Huygens deconvolution software package to generate theoretically derived, depth-variant point spread functions (PSFs) from the specified acquisition parameters (Fig. 1B, right)^8, 10^. In this approach, we specified 3 parameters to calculate a custom theoretical PSF: the lens immersion refractive index, the tissue embedding refractive index, and the distance from the coverslip to the start of the tissue. These theoretical PSFs were used across axial depths to deconvolve the image using the Huygens maximum likelihood estimation (MLE) function^12^. Deconvolution of the acquired image stacks was completed in under 5 minutes on our workstation and successfully removed out-of-focus light to resolve nuclei in the axial dimension (Fig. 1C). Overall, the imaging acquisition and processing of this 0.125 mm^3^ tissue volume was completed in 10-15 minutes and achieved nuclear resolution at a z-depth of 500 μm in the mouse brain cortex (Fig. 1C).

To further examine the axial resolution limit of this widefield approach and gauge the extent of time savings, we visualized pseudopod projections of brain macrophages from CX3CR1^ERCre^-tdTomato reporter mice (Fig. 1D, E). Deconvolution of microglia from imaging volumes acquired using the widefield microscope was even able to resolve thin microglial processes in the z dimension in addition to nuclei (Fig. 1D). Confocal imaging of the same tissue, with acquisition settings adjusted to match the pixel size and z-step size of the widefield system, took 2 hours and 50 minutes compared to the 15-minute total acquisition on the widefield system. While confocal microscopy achieved higher resolution of microglial processes (Fig. 1E), widefield imaging was more than 10 times faster and could still resolve major microglial pseudopod projections (Fig. 1D). We conclude that variant deconvolution can be applied to rapidly achieve subnuclear axial resolution with a widefield microscope in optically transparent tissue and has the potential to feasibly image large swaths of tissue at subnuclear resolution.

Our widefield approach was also useful in uncleared tissue, where structures of interest are near the tissue surface. We investigated the accumulation of β-amyloid (Aβ) in the adventitia of leptomeningeal arteries in an experimental mouse model of CAA driven by transgenic expression of mutant amyloid precursor protein and a knock-in of ApoE4 into the mouse ApoE gene locus (5XE4)^13^. In less than 30 min of acquisition and processing time, widefield images revealed the interspersed and interlocked nature of Aβ deposition between adventitial muscle cells in the leptomeningeal arterial tree (Fig. 1F), although without optical clearing, less than 100 μm of axial information could be resolved.

### Addressing technical limitations of deconvolution to facilitate large-volume imaging of pathology in 3D

After demonstrating nuclear and fine process resolution in Z with our widefield system, we next leveraged its speed to image large tissue volumes in 3D. Prior work imaging millimeters of human mesentery revealed key lymphatic features in Crohn’s disease^14^, but some samples required up to 72 hours or beyond on line-scanning confocal systems, limiting throughput. Prolonged acquisition also led to evaporation with water-immersion lenses, forcing the use of lower-resolution air objectives. To overcome these barriers, we implemented widefield z-stack stitching to rapidly extend imaging across large x-y areas, enabling scalable 3D tissue analysis.

**Figure 2.**
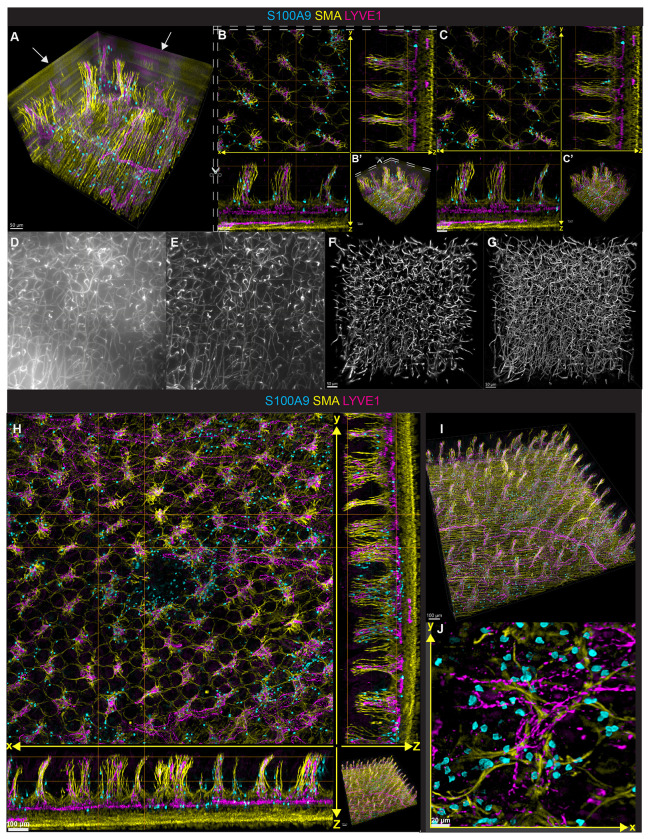
Optimizing deconvolution of deep tissue widefield imaging to enable tiling for large volume imaging (A) 3D display of single tile depicting striping edge artifacts (depicted by arrows) following deconvolution from mouse ileum immunostained with LYVE1 (magenta), smooth muscle actin (SMA, yellow), and S100A9 (cyan). (B) Extended display of same deconvolved tile as in (A) following cropping of artifacts as indicated with dashed lines and overview 3D display in (B’). (C) Extended display of the same tile in (A) when local background is subtracted before the deconvolution process without any cropping and a 3D overview (C’). (D) x-y projection of 142 microns depth of brain capillaries immunostained with anti-CD31 and anti-podocalyxin before background subtraction and (E) after subtraction of background found with a combined gaussian and minimum filter. (F) 3D projection of brain cortex following deconvolution of the whole volume as a single brick or (G) after subdividing the volume into 7 separate bricks in the Z axis for maximal retention of fine capillaries found in the raw image (D). (H) Extended display of a stitched 3x3 tile of mouse ileum after background subtraction and deconvolution with z bricks immunostained for LYVE1, SMA, and S100A9. (I) 3D display of entire overview from mouse ileum in (H). (J) x-y projection of 50 microns from ileum that depicts fine resolution of neutrophils in muscularis layer of mouse ileum from (H-I).

Applying default depth-variant deconvolution (Huygens) to individual widefield tiles introduced edge artifacts (Fig. 2A), the first challenge of deep imaging not previously observed in samples less than 10 μm thick^7^. While artifacts could be cropped out in isolated tiles (Fig. 2B, B’), this approach could not be used for large-scale reconstructions including greater axial depths. Artifacts were most prominent in channels that collected emission of Alexa Fluor 555 and Alexa Fluor 488, both typically prone to higher background, and rare in the channel collecting emission of Alexa Fluor 647, which exhibited low background. Most likely, tissue background levels were accentuated by extremely large PSF sizes in our thick tissue images not observed in typical sections that are less than or equal to 10 μm. To address this problem, we applied background subtraction using a small Gaussian kernel and a local minimum filter prior to deconvolution to correct for low-frequency variations, set below the spatial frequency of the PSF and image features, ensuring only true background was removed without compromising object signal. This correction eliminated scattered out-of-focus light without generating edge artifacts (Fig. 2C, C’). Raw images showed substantial background variability across single X-Y planes (Fig. 2D), which was normalized by subtraction (Fig. 2E). We hypothesize that the Huygens maximum likelihood estimation (MLE) algorithm misinterprets abrupt background shifts as large, undefined objects, projecting artifacts to volume edges. Background subtraction prior to deconvolution resolved this issue, enabling artifact-free recovery of in-focus signal.

The second challenge in applying Huygens depth-variant deconvolution to thick samples was the reduced ability of MLE to resolve dim signal across full Z-stacks of 500-μm depth (Fig. 2F). Unlike 10-μm sections, which contain a single layer of PSFs, thick stacks required deconvolution across dozens of interdependent layers. To address this challenge, we used the bricking function of Huygens, which was originally intended for computer memory-limited systems, to divide the stack into seven thick segments (i.e., “bricks”) in the z-dimension for separate deconvolution and subsequent automatic reassembly. This preserved true signal while removing scattered light (Fig. 2G).

We first validated our workflow by imaging a small region of the small intestine from a wild-type (WT) mouse. A custom Python script implemented our prefiltering strategy into Huygens for automated batch processing. Nine 3D tiles were deconvolved and stitched to reconstruct the intestinal images, revealing neutrophils and lymphatics in 3D (Fig. 2H, I). Indeed, pockets of neutrophils were recruited to intestinal villi in WT littermates without overt inflammation (Fig. 2H–J), a spatial pattern not easily appreciated in thin sections or confocal imaging of a singular location.

**Figure 3.**
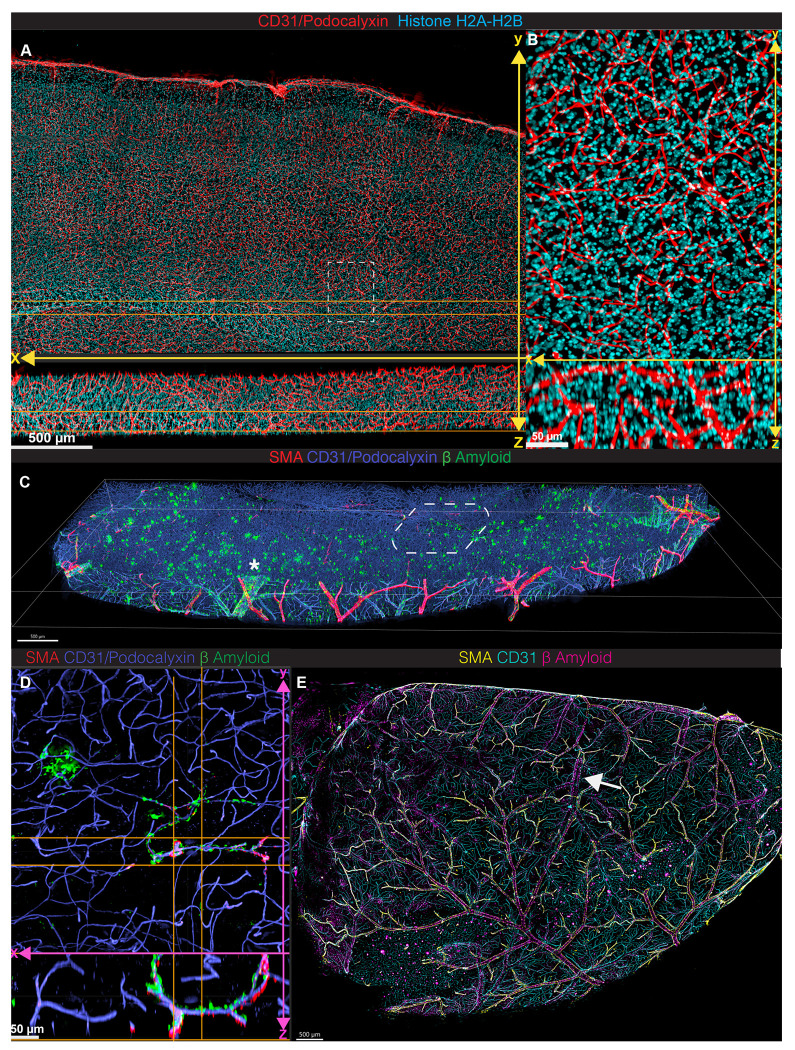
Large volume 3D widefield imaging enables comprehensive overview of CAA pathology at sub-nuclear resolution (A) Representative x-y projection of blood vasculature (CD31 and podocalyxin, red, and Histone-H3-ATTO488, cyan) from a 15-tile acquisition of a sagittal 500-micron section of mouse brain imaged, deconvolved, and stitched. (B) Extended display of image in panel (A) illustrating the depth and resolution of fine capillaries and nuclei. (C) Tilted 3D display of 80-tile image of Aβ plaques (green) in association with vessels (CD31/podocalyxin, blue) or arterioles (SMA, red) across sagittal section of brain isolated from 7-month old 5XE4 mouse acquired using 20X immersion lens, deconvolved, and stitched. * indicates bridging vein with deposits of Aβ plaques in SMA-negative vessel (i.e., bridging vein) accompanied by a SMA-positive vessel arteriole along the leptomeninges surface. (D) Extended display depicting three-dimensional CAA of capillaries zoomed in from (C, dashed box). (E) Maximum intensity projection after deconvolution of leptomeninges on the surface of an uncleared brain hemisphere from a 9-month old CAA mouse labeled for SMA (yellow), CD31 (cyan), and Aβ (magenta) and acquired using a 5X air objective (NA 0.16). Arrow indicates a bridging vein with Aβ deposition.

Expanding upon this proof-of-principle, we next imaged a large 3.75 [x] × 2.25 [y] mm area from a 500-μm cleared brain section stained for capillaries and nuclei. Composed of 15 stitched tiles, this image was acquired in under 1 hour on a widefield microscope (Fig. 3A), in contrast to the time of nearly 40 hours with line-scanning confocal microscopy at a similar pixel resolution. Deconvolved images resolved capillaries and nuclei across the full Z-depth, including near dense nuclear regions like the corpus callosum (Fig. 3B, Supplemental Video 1). This capability enabled acquisition of volumetric data over large tissue areas rather than isolated cross-sections.

3D imaging across wide X-Y areas better revealed spatial relationships that are lost in 2D. CAA is traditionally viewed in limited sections^13^, where leptomeningeal CAA plaques are observed as a thin line of staining at the edge of a brain section, and CAA along parenchymal vessels is often only observed in small fragments. Using our pipeline, we imaged a 1 cm^3^ volume of cortex from a 5XE4 mouse brain to a 500-μm depth in 5.5 hours (Fig. 3C, Supplemental Video 2). At sub-capillary Z-resolution, we visualized Aβ plaques along fine parenchymal vessels that would otherwise be missed in 2D (Fig. 3D). While the Aβ-laden vessels can be readily viewed in the 150-μm maximum intensity projection, the U shape of the vessel in the x-z view underscores that a standard 20-μm cross section would only show two lumina that would be challenging to recognize as CAA. We also visualized extensive CAA in the leptomeninges, including along vessels with low smooth muscle actin (SMA) signal (Fig. 3C).

The surface-exposed leptomeninges would be easiest to image without sectioning, but the curved dorsal surface of the brain has a range of at least 1.5 mm and cannot readily be peeled from the brain to prepare a flat mount as has been done previously to image lymphatics in the dura mater attached to the curved skull ^15^. We imaged the surface of an intact uncleared brain hemisphere using widefield microscopy in only 20 minutes (Fig. 3E, Supplemental Video 3). This approach revealed both expected Aβ deposition along SMA-positive arteries and novel deposition along SMA-negative bridging veins (Fig. 3E). These veins, identifiable by torn ends from dural detachment, are not typically implicated in CAA. However, their perivascular space was recently shown to allow CSF-derived solutes to enter the dura and access lymphatics ^16^, suggesting a role for bridging veins in Aβ clearance. These findings warrant further study into venous involvement in CAA.

In conclusion, we solved difficulties of applying Huygens depth variant deconvolution to very thick image volumes by first filtering out tissue background with high variance and then splitting the image into more manageable sections. Our solutions facilitated image processing of tiled image volumes as large as 1 cm^3^ while still achieving nuclear resolution in the z dimension. These larger 3D fields revealed previously unrecognized neutrophil distribution in the intestine of WT mice and captured 3D CAA features including previously unreported CAA along bridging veins. We also leveraged widefield imaging to rapidly image the surface of an uncleared brain hemisphere using our image processing pipeline to attain a global view of leptomeningeal CAA. Widefield 3D imaging is a fast and accessible approach to achieve overview-like perspectives while maintaining fine featured resolution to visualize pathology that is otherwise overlooked in standard 2D sections.

### Rapid optical transparency of human donor kidney in a time-constrained, clinical diagnostic context

**Figure 4.**
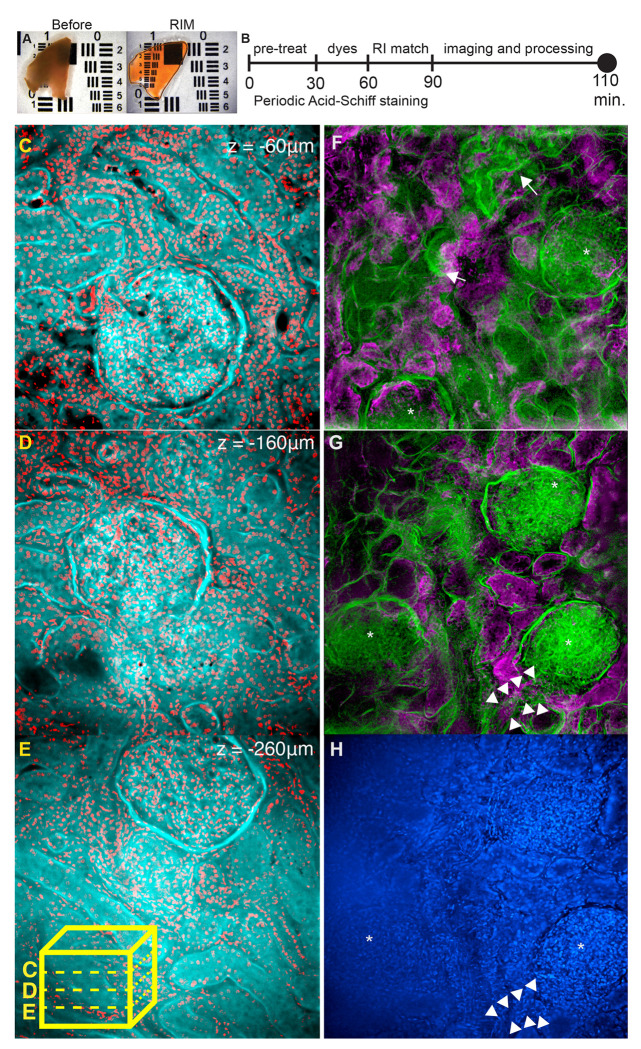
Rapid optical transparency and widefield imaging facilitates 3D evaluation of human kidney biopsies for pathology in a pre-transplant scenario (A) ~0.45 mm wedge biopsy treated with periodic acid, stained with CellBrite Orange, FAM-Hydrazide, and DAPI before refractive index matching (left) and after 30 minutes of refractive index matching (right). (B) Timeline overview to obtain optical transparency of wedge biopsy as seen in (A). (C-E) 5 μm X-Y maximum intensity projections of 3 separate glomeruli spaced at 100 μm z-intervals extracted from a single imaging volume of a kidney biopsy labeled with a Periodic Acid Schiff stain (cyan) and nuclei (red). Lower box schematic (yellow) of image volume from which digital sections were extracted at different depths. (F) Digital section from 3D image of biopsy constrained to timing shown in (B) depicting intact glomeruli and its neighboring proximal tubules, some of which contain atrophy (arrows). (G) 3 intact glomeruli (*), along with a possible arteriosclerotic vessel (arrowheads) found 100 microns of Z-depth below the digital section in (F) and its accompanying DAPI nuclear staining (H).

Rapid 3D imaging made possible by fast tissue processing and clearing paired with rapid image acquisition and processing described above, opens a path to clinical application of 3D imaging where time constraints are present. We considered the relevant clinical scenario where donor organs must be evaluated for transplant viability within a finite, defined time. Currently, the evaluation of donor kidney suitability for transplant requires pathologist evaluation of 5-μm frozen sections of a wedge biopsy collected from the putative donor organ. In a short time, while the donor organ is stored, the biopsy is evaluated to determine if it would provide a sufficiently healthy organ to warrant transplant. Considering that frozen sections inherently sample a small area of tissue, even a fraction of the biopsy collected, there may emerge undersampling error, with conclusions drawn that may not be representative of the actual status of the organ. Current standard of care is fraught with interobserver variability and significantly contributes to an overly high rate of discarding suitable tissues ^11^. 3D imaging of the biopsy might be a feasible alternative to current methodologies because it evaluates a larger portion of the organ that could lead to improved decision-making in the clinic. However, an obstacle to that potential solution is the challenge of carrying out a 3D evaluation within the defined time. To that end, we tested whether ADAPT-3D combined with a widefield imaging evaluation could lead to fast, informative pathological evaluation of human kidneys. Human kidney tissue from a mock wedge biopsy (Fig. 4A) measuring approximately 1 cm x 1 cm x 0.5 mm (x, y, z), equivalent to 150 individual frozen section slides, was fixed, stained with a fluorescent analog of the Periodic Acid-Schiff solution, and cleared with ADAPT-3D refractive index matching solution within 90 minutes and then imaged and processed using widefield microscopy and deconvolution in another 30 minutes (Fig. 4B).

To visualize glomeruli and its connecting tubules, a wedge biopsy stained with Periodic Acid Schiff and nuclear staining was imaged across over 300 μm of depth, followed by deconvolution. The resulting image volume captured three whole glomeruli at different depths (Fig. 4C–E), with 3D information allowing for clear distinction of closely situated glomeruli. By preserving overall structure without physical sectioning, we digital sliced through hundreds of consecutive z-planes, and visualized an afferent arteriole entering its corresponding glomerulus along with its associated proximal tubules at different depths (Supplemental Video 4). As we established the features of a relatively unremarkable biopsy, we applied this approach in a time-constrained manner to examine potential pathology as outlined in Fig. 4B. We found examples of atrophic tubules with thickened tubular basement membranes (Fig. 4E, F, arrows) and possible arteriosclerotic vessels (Fig. 4C, D, arrowheads). While optimization of this process for clinical utility and standards for evaluation would be needed, along with a collection of comparative studies evaluating 3D images versus frozen sections, these data indicate that tissue processing with a method like ADAPT-3D combined with a rapid method of image acquisition and analysis holds promise for 3D pathology in a time-constrained, clinically applicable setting.

In conclusion, this study reveals and optimizes how widefield microscopy can be coupled with depth-variant deconvolution and improved rapid tissue processing protocols to provide for much faster and more affordable 3D imaging. The financial aspects of epifluorescent microscope builds, objectives, computer and software, reduce costs substantially over confocal and light-sheet systems. Furthermore, whereas the latter typically require expensive service contracts, a service contract for the widefield system would not be necessary. For work in remote areas, including fieldwork, the widefield microscope build offers greater accessibility. Indeed, as clinical utility is considered, one might envision these types of microscopes on carts for use in operating rooms or clinical offices. For many laboratories, including our own, one might envision the advantages of this system to be one that does not always replace the utility of confocal or light-sheet fluorescence microscopy but does so on a select and even regular basis, such as times when higher throughput is critical, when water-dipping lenses are needed and evaporation over long imaging time would be an obstacle, and when the quality of the images on a widefield system meets the needs of a project. In busy groups where equipment is desired by several operators, time-saving options can be game-changing. We anticipate that, along with increasing computational power becoming ubiquitous, improvements to this pipeline will continue. Future efforts will focus on the ability to better deconvolve fluorescence signal where signal-to-noise is low and to evolve our protocol allow multiplex approaches. We also envision stitching in the z-axis to complement stitching in the x- and y-axes.

## Methods

### Mice

Studies in mice were approved by the Washington University Institutional Animal Care and Use Committee, protocol #22-0433 to GJR. All mice, including WT C57BL/6 mice, were bred in-house in Specific-Pathogen Free facilities. Other mouse strains were also maintained on the C57/BL6 background. 5xFAD (line 7031) bearing one or two human apoE4 alleles and zero or one mouse apoE allele that develop β-amyloid plaques characteristic of CAA and Alzheimer’s disease were obtained from David Holtzman (Washington University) with Materials Transfer Agreement between Cure Alzheimer’s Fund in place for the humanized apoE4 line with GJR. CX3CR1^ERCre^-tdTomato reporter mice were generated by crossing previously described CX3CR1^ERCre^ mice^17^ with TdTom^fl/fl^ mice^18^. TNF^ΔARE/+^ mice^19^ on were obtained from the Cleveland Clinic in 2016 and bred in-house.

### Human kidney biopsies and tissue processing

Human kidney samples were acquired from deceased donors whose kidneys were rejected for transplant through an agreement with Mid-America Transplant Foundation and associated IRB approval to J.P.G. For time-constrained processing of human kidneys, kidneys were biopsied into wedges first fixed in 4% paraformaldehyde (PFA) and stored until the trialed experiments. They were pre-treated in 0.5% periodic acid for 28 minutes, washed with 1X PBS containing 10 U/mL heparin, incubated in blocking buffer containing CellBrite Orange (Biotium, 1:200), FAM-Hydrazide (Lumiprobe, 15170, 1:100), and DAPI (Sigma-Aldrich, D9542, 1:200) for 25 minutes, washed with 1X PBS containing 10 U/mL heparin, and incubated in ADAPT-3D refractive index matching solution for 25 minutes before mounting the sample for imaging. The kidney tissue in Figure 4 without evident pathology was incubated in Decolorization/Delipidation Buffer with 0.2x PBS for 24 h, pretreated with periodic acid, stained with the same dyes for 1.5 h, and then incubated in refractive index matching solution for 1.5 h.

### Preparation and staining of tissues

The last two cm of the mouse ileum was collected after flushing the mouse small intestine with PBS and then was fixed in 4% (w/v) paraformaldehyde. For preparation of the sagittal brain sections, mouse brains were fixed in 4% w/v PFA at 4°C for 24 h, washed in PBS containing 10 U/mL heparin overnight, and sectioned on a LeicaVT1200 vibratome at 500-μm intervals. All subsequent steps were performed at room temperature. Sections were incubated in Decolorization/Delipidation Buffer with 0.2x PBS for 3-10 h, washed 3x 30 min with PBS containing 10 U/mL heparin, and then incubated in partial delipidation Buffer for 15-48 h. Samples were then washed 3x30 min in PBS with the first washed performed using 0.1X PBS and then blocked in ADAPT-3D blocking buffer for 3 h. Samples were stained for 15-36 h in blocking buffer containing antibodies against histone H2A-H2B heterodimers (Histone-Label Atto488, Chromotek, tba488, 1:200), CD31 (R&D Systems, AF3628, 1:200), Podocalyxin (R&D Systems, AF1556, 1:200), β-Amyloid (Clone D54D2, Cell Signaling Antibodies, 8243L, 1:200) conjugated to CF647 (Biotium Mix-n-Stain, 92259), and αSMA-AF488 (Clone 1A4, Sigma-Aldrich, 1:200). After 3x1 h washes in PBS with 0.2% tween 20 and 10U/mL heparin, thick brain sections were stained 1:200 with secondary antibodies prepared at 1.5mg/mL (Cy3 F(ab’)2 Donkey Anti-Sheep IgG, Jackson ImmunoResearch, 713-166-147), (Cy3 Donkey Anti-Rat IgG, Jackson ImmunoResearch, 712-165-153). Endogenous tdTomato signal in CX3CR1-tdTomato brain sections was boosted by staining with CF568 conjugated rabbit anti-RFP in blocking buffer (1:200, Biotium, 20477). Intestinal tissues were only processed with Decolorization/Delipidation Buffer before proceeding to the blocking step. Mouse ileum was stained with antibodies against alpha smooth muscle actin-Cy3 (clone 1A4, 1:200, Sigma-Aldrich, C6198) along with LYVE1 (Abcam, ab14917, 1:200) and S100A9 (Bio-techne R&D, AF2065, 1:200) that were conjugated to CF647 and CF488, respectively, using Mix-n-Stain^™^ Antibody Labeling Kit (Biotium, #92259, #92253) in ADAPT-3D blocking buffer. Antibody labeled tissues were incubated in ADAPT-3D refractive index matching solution diluted 1:1 with PBS for 30 minutes and then in full strength refractive index matching solution for 3 h directly prior to image acquisition.

### Image Acquisition: Epifluorescent and confocal microscopy

Zeiss Examiner Z1 was used as a base microscope and was fitted with a motorized x-y-z stage for fine control. The Colibri 7 LED light source was used to generate 7 different excitation lines, and a multiband pass filter set (Zeiss 112 HE) was used to collect emitted light without switching filter cubes. Images were acquired using either a 20X immersion lens (spherical aberration collar, NA 1.0) or 5X air lens (NA 0.16) with a Prime BSI express camera. All cleared tissues were imaged with the 20x lens immersed in water separated from the sample by a coverslip while uncleared tissues were submersed in PBS and imaged directly with either the 20x immersion or 5x air lens. Images were acquired at 0.8-1.2um z intervals with the 20x immersion lens and at 15um intervals with the 5x air lens in accordance with the Nyquist rate. For comparison images in Figure 1, images were taken on a Leica SP8 confocal microscope equipped with a 20X oil immersion lens (NA 0.75) with same pixel size as the 20X immersion lens.

### Image Processing: Deconvolution

Raw images were inputted into Huygens Professional software (Scientific Volume Imaging, Version 21.1.1) on a workstation with (Operating System: Windows 11 Pro, CPU: AMD Ryzen Threadripper PRO 5975WX 32-Core 3.6 GHz, GPU: NVIDIA GeForce RTX4090, RAM: 512 GB), For images acquired with the Zeiss epifluorescent microscope, .czi files were loaded into Huygens. Within the software, the image microscopic parameters were adjusted to the following: the lens embedding medium was set to refractive index of 1.33; the sample embedding medium was adjusted to refractive index of 1.50. The coverslip distance was adjusted by selecting the beginning of real signal in the coverslip distance editor with the imaging direction set to downward. Theoretical PSFs computed by the Huygens software were used for all deconvolutions. After parameterization, the background was found using a small Gaussian filter followed by a minimum filter and then subtracted from the original image in accordance with our supplemental code. Single tiles were deconvolved using the deconvolution wizard to create a deconvolution template. All images were deconvolved using the Quick Maximum Likelihood Estimation (QMLE) algorithm except for images of the human kidney which were deconvolved using the classic MLE algorithm. The signal to noise ratio (SNR) of unfiltered images was often 40 and above while the SNR of filtered images was lowered to 20 and below. Background was calculated on auto settings for unfiltered images but was manually set to zero for filtered images. Before starting the deconvolution, the acuity was either set to zero or increased to run more QMLE iterations such as on the images with microglial processes in figure 1. Bricking was set to ‘few bricks’ and GPU processing was enabled. For tiled images, each image was individually filtered and saved by running a custom python script in the Huygens python shell, and all images were deconvolved in the batch processor using parameter and deconvolution templates. The Huygens stitcher module was used to select a folder containing deconvolved images numbered by their order of acquisition, specify the image overlap and acquisition pattern, and the alignment between all tiles was calculated for all channels in x,y, and z dimension before stitching. For tiled images, image stacks were either corrected for vignetting using a flatfield reference or originally acquired with a cropped camera chip to reduce vignetting. All deconvolved images were saved as unsigned integer ICS2 file types. As a note, for the 80-tile brain scan, the required memory was roughly four times of the raw image file to both simultaneously load all 32-bit float type tiles and allocate space for the entire newly stitched image. As a workaround solution, virtual memory was allocated on the SSD C:/ drive.

### Image visualization

3D image volumes were visualized on Imaris software (Bitplane Inc.) on v10.1.1 software.

## Supplementary Material

1
