# Supplementary material for: Depth-Variant Deconvolution Applied to Widefield Microscopy for Rapid Large-Volume Tissue Imaging
Source: Res Sq. 2025 Jun 6:rs.3.rs-6710731. Preprint. [Version 1] doi: 10.21203/rs.3.rs-6710731/v1 (PMC12155202; doi:10.21203/rs.3.rs-6710731/v1)
Supplement: 1 [file NIHPPrs6710731v1-supplement-1.pdf]

Video 1 related to Figure 3. Multi-tile 3D imaging using epifluorescent widefield microscopy followed by deconvolution with depth-variant point spread functions. Sagittal sections from 9-month old mice were immunolabeled with traditional antibodies against CD31 and podocalyxin (red), and nanobodies against Histone H2A-H2B heterodimers (cyan).

Video 2 related to Figure 3. Deconvolved widefield (5X, NA 0.16) overview of CAA along leptomeningeal vessels across the entire dorsal surface of an intact 5XE4 brain hemisphere. Hemisphere of 9-month-old mice immunolabeled with CD31 (cyan), alpha-smooth muscle actin (yellow), and beta-amyloid plaques (magenta). Plaques are evident along both middle cerebral artery (first zoom-in) and bridging vein (second zoom-in).

Video 3 related to Figure 3. Widefield 3D volume of entire mouse cortex contained in a 500 μm sagittal section deconvolved to achieve high axial resolution. Sagittal sections of brains from CD31 and Podocalyxin (blue), alpha-smooth muscle actin (red), and beta-amyloid plaques (green). Plaque deposition can be seen not only along arterioles but also in both bridging veins of leptomeninges and vessels throughout cortex.

Video 4 related to Figure 4. 3D imaging by epifluorescent widefield microscopy and deconvolution allows for rapid, continuous axial examination of human kidney wedge biopsies. Fly through of human kidney 3D imaging volume viewed in 5 µm digital sections labeled with a fluorescent Periodic Acid Schiff stain (cyan) and nuclear DAPI staining (red). Magenta arrow indicates arteriole entering glomeruli and magenta circle highlights the connecting proximal tubule in 3D.
